# Co-Infection with the Friend Retrovirus and Mouse Scrapie Does Not Alter Prion Disease Pathogenesis in Susceptible Mice

**DOI:** 10.1371/journal.pone.0030872

**Published:** 2012-01-25

**Authors:** Pascal Leblanc, Kim Hasenkrug, Anne Ward, Lara Myers, Ronald J. Messer, Sandrine Alais, Andrew Timmes, Sue Priola

**Affiliations:** 1 Université de Lyon, Lyon, France; 2 Laboratoire de Virologie Humaine, INSERM U758, Lyon, France; 3 Laboratoire de Biologie Moléculaire de la Cellule, UMR5239, Lyon, France; 4 Ecole Normale Supérieure de Lyon, Lyon, France; 5 Laboratory of Persistent Viral Diseases, Rocky Mountain Laboratories, National Institute of Allergy and Infectious Diseases, National Institutes of Health, Hamilton, Montana, United States of America; Ohio State University, United States of America

## Abstract

Prion diseases are fatal, transmissible neurodegenerative diseases of the central nervous system. An abnormally protease-resistant and insoluble form (PrP^Sc^) of the normally soluble protease-sensitive host prion protein (PrP^C^) is the major component of the infectious prion. During the course of prion disease, PrP^Sc^ accumulates primarily in the lymphoreticular and central nervous systems. Recent studies have shown that co-infection of prion-infected fibroblast cells with the Moloney murine leukemia virus (Mo-MuLV) strongly enhanced the release and spread of scrapie infectivity in cell culture, suggesting that retroviral coinfection might significantly influence prion spread and disease incubation times in vivo. We now show that another retrovirus, the murine leukemia virus Friend (F-MuLV), also enhanced the release and spread of scrapie infectivity in cell culture. However, peripheral co-infection of mice with both Friend virus and the mouse scrapie strain 22L did not alter scrapie disease incubation times, the levels of PrP^Sc^ in the brain or spleen, or the distribution of pathological lesions in the brain. Thus, retroviral co-infection does not necessarily alter prion disease pathogenesis in vivo, most likely because of different cell-specific sites of replication for scrapie and F-MuLV.

## Introduction

Prion diseases, also known as transmissible spongiform encephalopathies (TSEs), are fatal, neurodegenerative diseases that include Creutzfeldt-Jakob disease (CJD) in humans, scrapie in sheep, bovine spongiform encephalopathy (BSE) in cattle, and chronic wasting disease in deer and elk. Prion diseases are characterized by the conformational conversion of the normally soluble, protease-sensitive cellular prion protein (PrP^C^) into an abnormal detergent-insoluble and partially protease resistant isoform called PrP^Sc^. PrP^Sc^ has been found to be tightly associated with prion infectivity and recent evidence has shown that PrP^Sc^ is the major component of the prion infectious agent [1], [2].

PrP^C^ is a cell-surface glycoprotein expressed in various mammalian tissues including brain, spinal cord, nerve, heart, muscle, spleen and lymph node (for review see [3]). Thus, even though the major pathological hallmarks of prion disease are spongiform degeneration and gliosis in the brain, prion infection and the conversion of PrP^C^ to PrP^Sc^ are not necessarily restricted to brain areas but can also occur in peripheral lymphoid tissues such as spleen and lymph nodes [4]. The conversion of PrP^C^ to PrP^Sc^ [5] and the spread of the prion agent from peripheral sites of infection to the brain and vice versa [4], [6]–[10] are key events in the pathogenesis of prion diseases. However, in some pathological contexts such as inflammation, prion infectivity and PrP^Sc^ can be found in tissues that are not normally associated with prion infection [11]–[13]. Indeed, chronic inflammatory conditions such as nephritis, hepatitis or mastitis can lead to changes in the distribution of scrapie infectivity in the organism although the mechanisms involved are poorly understood [11]–[13].

Recently, we found that co-infection of scrapie-infected mouse fibroblast cells with the Moloney Murine Leukemia retrovirus (Mo-MuLV) strongly enhanced the release and spread of scrapie infectivity in cell culture. Specifically, we found that PrP^Sc^ and infectivity were associated with Mo-MuLV viral particles as well as exosomes and observed that Mo-MuLV infection strongly stimulated the release of exosomes [14]. In agreement with our findings, the small-ruminant caprine arthritis encephalitis lentivirus (CAEV) was also found to enhance PrP^Sc^ accumulation in co-infected, cultured sheep microglial cells as well as culture supernatant [15]. Similarly, Ligios et al. showed that the lentivirus Maedi Visna virus, a common cause of lymphofollicular mastitits in sheep, leads to an inflammatory response which is associated with an increase in prion propagation and secretion of prions into milk [13]. Taken together, the data suggest that retroviral co-infection might facilitate the spread of prions in vivo by significantly increasing the level of PrP^Sc^ released from infected tissues. If this hypothesis is correct, then peripheral co-infection of a mouse with scrapie and the Mo-MuLV retrovirus would lead to a more rapid spread of scrapie infectivity and shorter scrapie disease incubation times.

When inoculated peripherally, the most commonly used strains of mouse scrapie have disease incubation times in excess of 6 months. Mo-MuLV is a highly pathogenic mouse retrovirus which causes a fatal T-cell lymphoma 3–5 months after inoculation into susceptible newborn mice [16]. Thus, co-infected mice might succumb to T-cell lymphoma before they became clinically ill with scrapie. To address the issue of whether or not retroviral infection of a mouse would alter prion pathogenesis, murine Friend leukemia retrovirus (F-MuLV) was substituted for Mo-MuLV. F-MuLV and spleen focus-forming virus (SFFV) together form the pathogenic Friend virus complex (FV) [17] which infects adult immunocompetent mice [18]. Co-infection with mouse scrapie and FV has been done before using mice highly susceptible to Friend virus-induced leukemia [19] with no effect on scrapie. However, this lack of effect might have been due to lack of virus early during prion infection since the mice were infected with FV months after being infected with murine scrapie. In order to ensure that actively replicating virus would be present for the entire course of prion infection, we used a mouse strain, (C57BL/10× A.BY)F1, that becomes highly viremic during the first weeks of acute FV infection but which then mounts strong immune responses and recovers without the development of leukemia [18]. Importantly, following the immune-mediated resolution of the acute FV infection, infectious virus still persists at detectable levels in the spleen for the lifetime of the mouse [20]. Thus, unlike the previous study [19], actively replicating retrovirus would be present at high levels during the early stages of mouse prion infection where peripheral prion replication and spread can be critical for efficient neuroinvasion [6]–[10] as well as at low levels in the spleen throughout the rest of the scrapie incubation period.

In the current study we show that, similarly to Mo-MuLV, co-infection of mouse scrapie-infected tissue cells with F-MuLV leads to an increase in exosomes, prion protein and scrapie infectivity in vitro. However, peripheral co-infection of mice with 22L mouse scrapie and FV did not lead to a decrease in scrapie disease incubation times or any significant alterations in 22L scrapie pathogenesis. Our results show that retroviral co-infection does not necessarily alter prion disease pathogenesis in vivo.

## Materials and Methods

### Cells

Mouse NIH3T3-22L fibroblasts infected with the 22L scrapie strain were previously described in Vorberg et al. [21] while NIH3T3-22L/MoMuLV were previously described in Leblanc et al. [14]. The neuroblastoma cells N2a#58 and N2a#22L were kindly provided by Sylvain Lehmann and were described in Nishida et al. [22]. NIH3T3-22L/FB29 and NIH3T3-22L/57 are mouse fibroblast cells co-infected with the mouse scrapie strain 22L and the Friend murine leukemia virus (F-MuLV) strains FB29 or Fr57, respectively. NIH3T3 and N2a cells were maintained in DMEM or OptiMEM medium (Invitrogen) supplemented with 10% FCS, L-glutamine and penicillin/streptomycin, respectively.

### Antibodies

Monoclonal anti-PrP antibodies SAF-32 and SAF-83 were obtained from J. Grassi (CEA Saclay, SPIbio), 6D11 was from Covance Research Products and D13 from InPro Technology. The Rabbit a-Envgp70 (DJ-39462) and a-CAp30 (DJ-39461) antibodies were kindly provided by RJ Gorelick (AIDS Vaccine program, SAIC-Frederick). The anti-EF1α antibody (clone CBP-KK1, ref 05-235) was purchased from Upstate cell signaling.

### Reverse Transcriptase detection

Quantification of virus production was assessed by measuring virus associated Reverse Transcriptase activity (RT) released in the cell culture supernatant. Briefly, 10 µl of culture supernatant was added to 50 µl of a reverse transcription mix (60 mM Tris-Cl (pH 8), 75 mM NaCl, 0.06% Nonidet P-40 substitute (Sigma), 0.7 mM MnCl_2_, 6 mM dithiothreitol, 6 µg·ml^−1^ oligo(dT), 12 µg·ml^−1^ poly(rA), and 2 µCi·ml^−1^ [α-^32^P]dTTP). After 1 h of incubation at 37°C, 10 µl was spotted onto DEAE membrane (DE-81; Whatman), washed 3×10 minutes with 2× SSC (0.3 M NaCl, 0.03 M sodium citrate [pH 5]) and rinsed with 100% ethanol. The membrane was exposed to a phosphorscreen (Molecular Dynamics) and radioactive synthesized cDNAs were measured with a phosophorimager (Fuji), and quantified by using MultiGauge software (Fuji).

### Western blotting

NIH3T3-22L and NIH3T3-22L/F-MuLV cells were recovered and lysed in sample buffer and boiled 5 min at 95°C. Total protein (15 µg) was analyzed by SDS-PAGE and proteins were transferred onto PVDF membrane (Amersham) in Tris-glycine buffer containing 20% methanol for 1.5 h at 2 mA/cm2. Membranes were blocked for 30 min with 5% milk in TTBS (Euromedex) and probed with antibodies directed against Envgp70, CAp30, PrP or EF1α. After extensive washing in TTBS, membranes were incubated with conjugated-peroxidase secondary antibodies for 1 h and then washed for 30 min. Proteins were detected by the enhanced chemiluminescence method (Super Signal West Pico, Pierce).

### Differential centrifugation

Cell culture supernatants from NIH3T3-22L, NIH3T3-22L/MoMuLV and NIH3T3-22L/F-MuLV cells were centrifuged 5 min at 3000 g and 5 min at 4500 g to remove cells in suspension, ultracentrifuged 30 min at 10000 g to remove cellular debris and finally centrifuged at 100000 g for 1 h to pellet virions and exosomes. The resulting pellets were directly resuspended in phosphate buffer saline (PBS) and directly loaded on N2a#58 target cells or resuspended in sample buffer and analyzed by Western blotting using anti-Envgp70, anti-CAp30, anti-PrP or anti-EF1α antibodies.

### Coculture experiments

N2a#58 target cells were plated at a density of 2.5×10^5^ cells on the growth surface of a six-well plate. For insert co-cultures, NIH3T3-22L, NIH3T3-22L/MoMuLV and NIH3T3-22L/F-MuLV cells were plated at 2.5×10^5^ cells onto the growth surface of a 4.2 cm^2^ membrane with high density pores of 0.4 mm diameter (Becton Dickinson). Contact between cells was for 4 days followed by passage of the target cells for 2 weeks (5 passages).

### Cell blot assay

To detect the proteinase K (PK) resistant PrP, N2a#58 target cells were transferred and blotted onto a nitrocellulose membrane using a homemade cell blot apparatus. Detection of PrP^Sc^ was performed as previously described [14], [23]. The PK-treated membrane was analyzed using a mixture of antibodies (SAF32+SAF83) and a secondary antibody labelled with HRP (Dako). N2a#58 and scrapie infected N2a#22L were used as negative and positive controls for PrP^Sc^, respectively.

### Co-infection of mice

The F1 cross of C57BL/10× A.BY mice [(B10 X A.BY)F1] were first infected intravenously (i.v.) via the retro-orbital route with 500 µl of a lactate dehydrogenase-elevating virus-free stock of Friend virus complex (FV) [18] which had been diluted 1∶10 in PBBS:2% FBS. The titer of the FV complex was 2×10^5^ FFU/ml of F-MuLV and 2×10^3^ SFFU/ml of spleen focus-forming virus. Immediately following the F-MuLV inoculation, (B10 X A.BY)F1 mice were inoculated intraperitoneally (i.p.) with 50 ul of a 10% 22L brain homogenate stock diluted to 1% in PBBS:2% FBS. The i.p. titer of the 22L brain homogenate used was 2×10^5.3^ ID_50_/g. Rocky Mountain Laboratories (RML) is an Association for Assessment of Laboratory Animal Care (AALAC)-accredited facility and all animal procedures were carried out in strict accordance with the recommendations in the Guide for the Care and Use of Laboratory Animals of the National Institutes of Health (NIH). The protocol used was approved by the RML Animal Care and Use Committee and the NIH (protocol #2006-34). All procedures were done under isoflurane anesthesia and all efforts were made to minimize distress.

### Quantification of tissue PrP-res levels

Dilutions of a pool of 22L+FV brain homogenate were used as a standard curve to determine the relative PrP^Sc^ content of individual infected mice. Briefly, brains were homogenized in phosphate buffered saline (PBS) by probe sonication for 10×10 secs on ice. Homogenates were diluted to 0.1% brain in PBS and digested with 20 µg/ml PK at 37°C for 60 min. PK digested samples were separated by SDS-PAGE and transferred to PVDF membrane for western blot analysis using the anti-PrP monoclonal antibody 6D11 (1∶5,000) followed by an anti-mouse IgG horseradish peroxidase conjugated sheep secondary antibody (1∶400,000, GE Healthcare) and chemiluminescent development using the ECL Advance kit (GE Healthcare). All film exposures were scanned using a Microtek scanner at 600 dpi in grayscale mode. The resulting tiff image was converted to a gel file and rectangles were drawn on the image to encompass all three PrP^Sc^ glycoforms in each lane along with a rectangle in an adjacent blank area to determine background. The signal intensity in each lane was quantified using the program ImageQuant 5.2 and the resultant data were plotted in Excel. The Trendline function was used to fit a curve to the data and yield an equation for interpolating points. The Logarithmic function gave the best fit with R^2^ values>0.99.

### Histology

Paraffin-embedded mouse brain and spleen sections were de-paraffinized and stained with hemotoxylin and eosin. For detection of PrP^Sc^, slides were immersed in 10 mM sodium citrate buffer pH 6.0 at 120°C/20psi for 20 minutes (hydrated autoclaving). Slides were exposed overnight at 4°C to the anti-PrP antibody D13 diluted 1∶500 in 1% normal goat serum. Secondary antibody was biotinylated anti-human IgG (Jackson Laboratories) diluted 1∶500 in 1% normal goat serum and was applied on a Ventana NexES automated stainer and developed using alkaline phosphatase conjugated streptavidin (Biogenex) and the Ventana Fast Red chromagen.

### Flow cytometric analysis

Four (B10 X A.BY)F1 mice were inoculated retro-orbitally with FV as described above. At 7 days post-infection, follicular dendritic cells (FDCs) were obtained from spleens dissociated in a 6 well plate with a 100 µm nylon mesh cell strainer (Fisher Scientific) in Minimal Essential Medium (MEM, Invitrogen) plus 150 U/ml collagenase (Gibco), 1 Mm CaCl_2_ and 1 Mm MgCl_2_. After a one hour incubation at 37°C, samples were transferred to a 50 ml conical tube and washed with MEM. Red blood cells were lysed with ammonium chloride TRIS. The antibodies used for cell staining were: Pacific Blue anti-NK1.1(PK136) (Biolegend); Pacific Orange anti-CD4; FITC anti-CD3; PE anti-Gr-1; PE-Texas Red anti-CD45R/B220 (RA3-6B2); PerCp-Cy5.5 anti-CD19 (ID3); PE-Cy7 anti-CD11c (HL3); A700 anti-CD11b (M1/70); APC-Cy7 anti-CD8 (53-6.7); biotin anti-I-Ap followed by Qdot605 anti-SAv (Molecular Probes), which were purchased from BD Pharmingen, eBioscience or Invitrogen unless otherwise noted. To detect FV infection, cells were first incubated with an antibody to the F-MuLV glycosylated gag protein (mAb 34, hybridoma supernatant), followed by Cy5-goat-anti-mouse IgG2b [18]. The data were collected using an SORP LSRII (BD Biosciences) flow cytometer and were analyzed using FlowJo (Tree Star). The gating strategy for the representative flow cytometric dot plot was a live gate negative for CD11c, CD8, CD19, CD4, CD3, CD11b, Gr-1, and positive for MHC class II and B220 as shown.

## Results

### F-MuLV co-infection of 22L scrapie-infected NIH 3T3 cells

**Figure 1:**
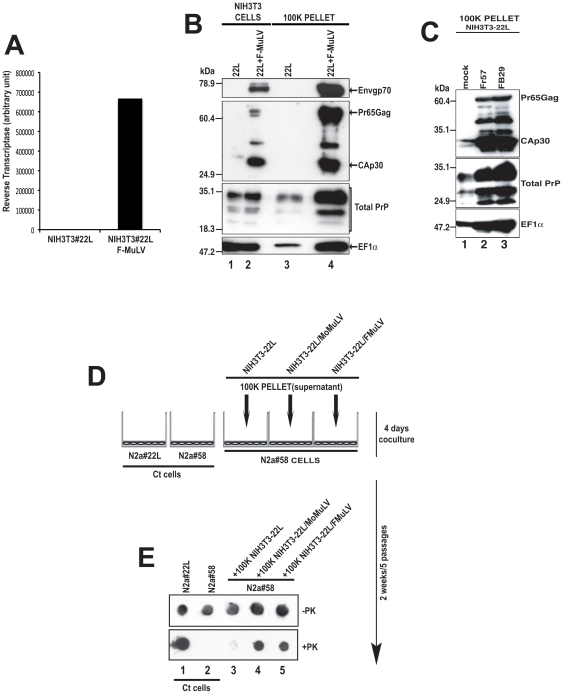
The Friend Murine Leukemia Virus (F-MuLV) strongly enhances PrP levels and prion infectivity released into the cell culture supernatant. (**A**) Reverse transcriptase (RT) levels in NIH3T3-22L cells alone (22L) and NIH3T3 cells coinfected with the F-MuLV strain FB29 (22L+F-MuLV). (**B**) NIH3T3-22L (lane 1) and NIH3T3-22L/F-MuLV cells (lane 2) were assayed for F-MuLV expression using the DJ-39462 antibody for the viral envelope (Envgp70, Panel 1) and the DJ-39461 antibody for the viral capsid (CAp30, panel 2). PrP was detected using SAF-32 (Total PrP, panel 3) and exosomes using anti-EF1α (panel 4). The proteins recognized are indicated on the right side of the figure. Molecular mass markers are shown on the left side of the panel and lane numbers are indicated on the bottom of the figure. (**C**) NIH3T3-22L cells (mock) or NIH3T3-22L cells co-infected with the Friend viruses Fr57 or FB29 were analyzed by western blot for F-MuLV expression as well as PrP and exosome release as in panel B. Note that the F-MuLV Fr57 strain, like FB29, strongly enhances PrP and exosome release. Molecular mass markers are shown on the left side of the panel and lane numbers are indicated on the bottom of the figure. The proteins recognized are indicated on the right side of the figure. (**D**) Coculture assay. Uninfected target N2a#58 neuroblastoma cells grown on the bottom surface of a six well plate and a 0.4 mm pore size insert containing the 100 K pellet from the supernatants of NIH3T3-22L, NIH3T3-22L/MoMuLV or NIH3T3-22L/F-MuLV were cocultured for 4 days. The N2a#58 target cells were then passaged 5 times over 2 weeks and assayed for PrP^Sc^. Ct = control. (**E**) Total PrP (-PK) and PrP^Sc^ (+PK) levels in N2a58 cells co-cultured with NIH3T3-22L/MoMuLV or NIH3T3-22L/F-MuLV supernatants were analyzed by dot blot. Ct cells = 22L infected and uninfected N2a#58 cells not exposed to supernatant. Lane numbers are shown at the bottom of the panel.

Prior to co-infecting mice with both scrapie and retrovirus, we first needed to determine whether or not F-MuLV, like MoMuLV, could enhance the release of prion protein and scrapie infectivity in scrapie infected tissue culture cells. NIH3T3 cells infected with the mouse scrapie strain 22L (NIH3T3-22L cells) were co-infected with the F-MuLV strain FB29 (NIH3T3-22L/F-MuLV cells). Viral replication was monitored ten days after infection by measuring the reverse transcriptase activity in the culture medium and assaying expression of the viral capsid protein CAp30, the Gag polyprotein precursor Pr65Gag and the envelope glycoprotein Envgp70. As shown in Figure 1, NIH3T3-22L cells co-infected with F-MuLV demonstrated significant virus-associated reverse transcriptase activity (Figure 1A) and expressed all three viral structural proteins (Figure 1B). These data show that F-MuLV replicated in NIH3T3-22L cells.

### Increased PrP levels and prion infectivity in the medium of NIH3T3-22L/F-MuLV cells

To determine if F-MuLV strongly enhanced the release of prion protein into the cell culture medium, supernatants from NIH3T3-22L cells and NIH3T3-22L cells co-infected with F-MuLV were recovered and subjected to differential centrifugation. The final pellets were analyzed by western blotting using antibodies directed against PrP, CAp30/Pr65Gag and Envgp70. As shown in Figure 1B, F-MuLV infection strongly stimulated the release of prion protein as judged by the increased amount of PrP in material pelleted from the medium of co-infected cells (Figure 1B, compare lanes 3 and 4). Additionally, we found that F-MuLV significantly increased the release of exosomes into the cell supernatant as judged by the increased amount of the EF1α exosome marker in the pellet derived from the centrifuged medium of co-infected cells (Figure 1B, compare lanes 3 and 4). Similar data were obtained using the F-MuLV strain Fr57 (Figure 1C). Thus, just as with Mo-MuLV [14], F-MuLV co-infection of NIH3T3-22L cells stimulated the release of prion protein and exosomes into the cellular supernatant.

To determine if the increased level of PrP in the cellular medium of NIH3T3-22L/F-MuLV cells correlated with an increase in scrapie infectivity in the medium, scrapie-susceptible murine neuroblastoma cells (N2a#58) were exposed to 100 K centrifugation pellets from the medium of either NIH3T3-22L or NIH3T3-22L/F-MuLV cells. As a positive control, 100 K pellets from the medium of NIH3T3-22L cells co-infected with Mo-MuLV (NIH3T3-22L/Mo-MuLV cells) [14] were also tested. After 4 days of coculture, the N2a#58 cells were passaged 5 times to eliminate the starting inoculum, the cells were harvested, and the presence of PrP^Sc^ was determined using the cell blot assay (Figure 1D) [14]. As shown in Figure 1E, in the absence of PK treatment, all cells expressed similar levels of PrP. However, after PK treatment, N2a#58 cells co-cultured with the 100 K pellets from the medium of either NIH3T3-22L/F-MuLV or NIH3T3-22L/Mo-MuLV cells expressed higher levels of protease-resistant PrP than cells infected with 22L alone. These results indicated that co-infected cells contained higher levels of scrapie infectivity in the cell medium than cells infected with 22L alone. Thus, F-MuLV acted similarly to Mo-MuLV [14] in vitro and strongly stimulated the release of PrP, exosomes, and scrapie infectivity into the cell culture medium of mouse scrapie-infected cells.

### FV and 22L scrapie co-infection of susceptible mice

**Figure 2:**
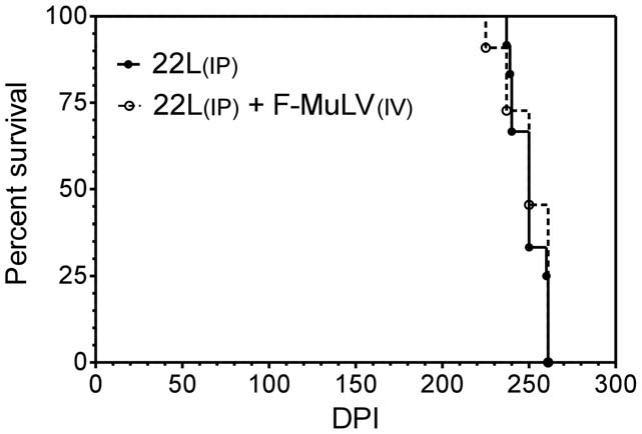
Co-infection of mice with 22L scrapie and F-MuLV does not affect scrapie disease incubation time. Survival curve of (B10 X A.BY)F1 mice inoculated intraperitoneally (i.p.) with 22L mouse scrapie alone (closed circles) or co-infected with 22L scrapie i.p. and FV intravenously (i.v., open circles). dpi = days post-infection.

In order to determine whether or not retroviral co-infection could affect scrapie disease incubation times, (B10 X A.BY)F1 mice were inoculated i.p. with the 22L strain of mouse scrapie and i.v. with a pathogenic retrovirus stock containing a complex of F-MuLV helper virus and spleen focus-forming virus known as Friend virus complex (FV) [18]. These mice support high levels of virus replication but then recover and maintain long-term persistent virus for life [24]. As expected, control mice inoculated with FV alone recovered from retroviral infection with the only clinical sign being moderate splenomegaly (data not shown). Control (B10 X A.BY)F1 mice inoculated i.p. with 22L scrapie all developed clinical scrapie with an average disease incubation time of 250±9 days. Mice inoculated with both FV and 22L scrapie also had moderate splenomegaly post-infection and developed clinical scrapie after 250±13 days. Thus, there was no significant difference in mean survival times between the two groups (Figure 2). These data show that i.v. retroviral co-infection did not affect scrapie disease incubation times.

### PrP^Sc^ levels and distribution in mice co-infected with 22L mouse scrapie and FV

**Figure 3:**
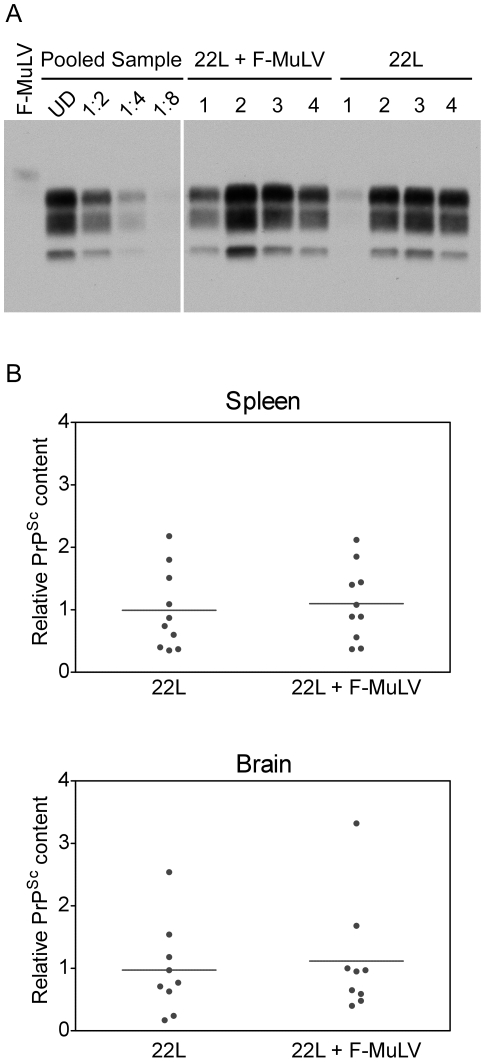
PrP^Sc^ accumulates to equivalent levels in the brain and spleen of mice infected with 22L or 22L plus F-MuLV. (A) Western blot of PK-treated spleen homogenates from (B10 X A.BY)F1 mice either inoculated i.v. with FV alone (F-MuLV), i.p. with mouse 22L mouse scrapie alone (22L), or co-infected with 22L mouse scrapie i.p. and FV i.v. (22L+F-MuLV). Samples from 4 individual mice are shown for both 22L and 22L+FV and a single sample for FV infected mice. Lanes 2–5 show dilutions of a standard comprised of equal amounts of spleen homogenate from individual mice inoculated with 22L+FV (Pooled Sample) used to calculate the relative amount of PrP^Sc^ in the spleen of infected mice. (B) The relative amount of PrP^Sc^ in each lane in (A) was determined by quantitative comparison to a standard curve derived from equal amounts of spleen homogenate (as shown in panel A) or brain homogenate (data not shown) derived from individual mice inoculated with 22L+FV.

Although the scrapie incubation period was not affected, it remained possible that co-infection altered the level of PrP^Sc^ accumulation. The amount of PrP^Sc^ present in the brains and spleens of co-infected mice was compared to the amount of PrP^Sc^ in the brains and spleens of mice infected with 22L alone. As shown in Figure 3A, the amount of PrP^Sc^ present in the spleen varied within each group, with some mice having higher levels of PrP^Sc^ in the spleen at late-stage clinical disease than others. When the amount of PrP^Sc^ was quantified, no significant mean difference was seen between mice infected with 22L alone versus mice co-infected with 22L and FV (Figure 3B). Similarly, there was no difference between the two groups of mice in the amount of PrP^Sc^ present in the brain (Figure 3B). These results are consistent with the similar disease incubation times observed for mice infected with 22L alone or mice infected with both 22L and FV and suggest that FV co-infection does not lead to an increased accumulation of PrP^Sc^ during mouse scrapie infection.

**Figure 4:**
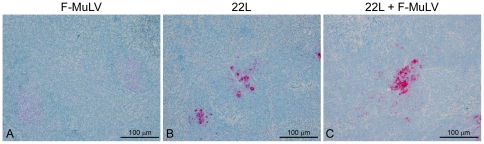
PrP^Sc^ localization in the spleen is the same in mice infected with 22L or 22L plus FV. PrP^Sc^ in spleens from (B10 X A.BY)F1 mice inoculated i.v. with FV alone (A), i.p. with mouse 22L mouse scrapie alone (B), or co-infected with 22L mouse scrapie i.p. and FV i.v. (C). Sections were stained using the anti-PrP mouse monoclonal antibody D13. Magnification = 200×.

While replication of FV in the spleen did not appear to increase the level of PrP^Sc^ in the spleen, it was possible that the distribution of PrP^Sc^ was altered. Spleens from three mice infected either with 22L plus FV, 22L alone, or FV alone were stained with the anti-PrP antibody D13 and the distribution of PrP^Sc^ analyzed. Representative results are shown in Figure 4. In (B10 X A.BY)F1 mice inoculated with 22L alone, punctate deposits of PrP^Sc^ were found primarily in the white pulp in a pattern consistent with deposition in follicular dendritic cells (Figure 4B). A similar pattern of PrP^Sc^ deposition was found in mice co-infected with 22L and FV (Fig. 4C), suggesting that FV co-infection did not lead to a change in PrP^Sc^ distribution in the spleen.

**Figure 5:**
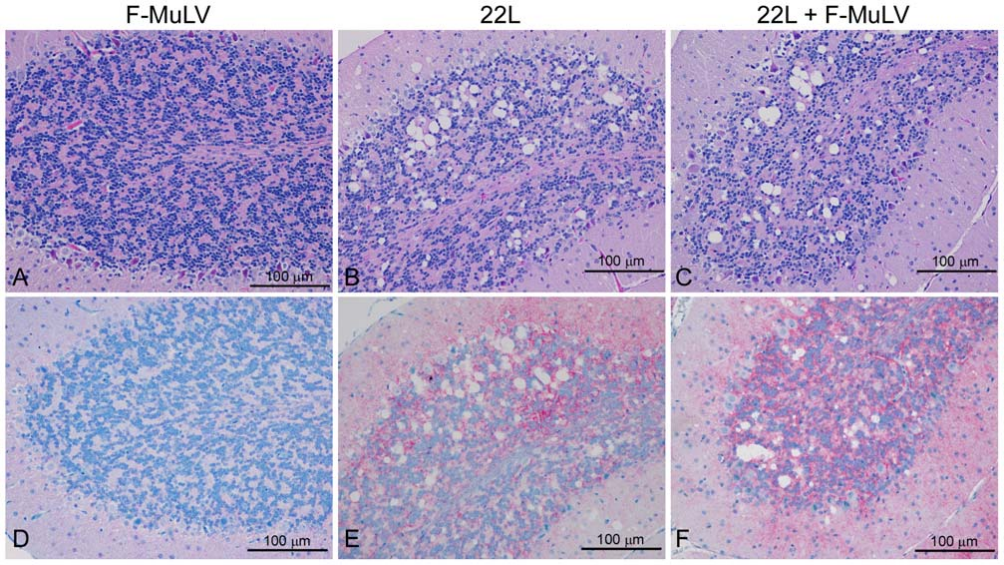
Vacuolation and PrPS^c^ deposition in the brain is the same in mice infected with 22L or 22L plus FV. (A–C) Hemotoxylin and eosin staining of the cerebellum from (B10 X A.BY)F1 mice inoculated (A) i.v. with FV alone, (B) i.p. with mouse 22L mouse scrapie alone, or (C) co-infected with 22L mouse scrapie i.p. and FV i.v.. Magnification = 200×. (D–F) PrP^Sc^ deposition in the cerebellum of (B10 X A.BY)F1 mice inoculated (D) i.v. with FV alone, (E) i.p. with mouse 22L mouse scrapie alone, or (F) co-infected with 22L mouse scrapie i.p. and FV i.v.. Sections were stained using the anti-PrP mouse monoclonal antibody D13 following hydrated autoclaving at high pressure and temperature. Red stain indicates the presence of PrP^Sc^. Magnification = 200×.

Although retroviral co-infection did not affect either scrapie disease incubation times or PrP^Sc^ levels at the clinical stage of disease, it could have affected the pathology induced by scrapie infection in the brain. The major pathological hallmark of the 22L strain of mouse scrapie is extensive vacuolation in the cerebellum [25]. In order to determine whether or not there was a difference in cerebellar vacuolation in (B10 X A.BY)F1 mice co-infected with 22L and FV, the extent of vacuolation in the brains of three co-infected mice, 22L infected mice, or FV infected mice were analyzed. Representative results are shown in Figure 5. When compared to mice infected with 22L scrapie alone, no difference was seen in the extent or localization of vacuolation in the cerebellum (Figure 5A–C) or other regions of the brain (data not shown) of mice co-infected with 22L and FV. Similarly, no differences in the distribution of PrP^Sc^ were observed in the cerebellum (Figure 5D–F) or other brains areas (data not shown) between control mice infected with 22L alone and mice co-infected with 22L and FV. Thus, retroviral co-infection of 22L scrapie-infected mice did not noticeably alter the pathology of 22L scrapie in the brain.

### Spleen follicular dendritic cells and FV infection

**Figure 6:**
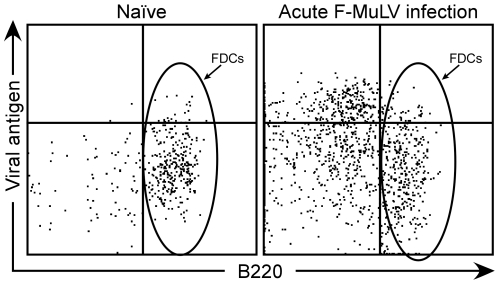
Analysis of follicular dendritic cells (FDCs) for Friend virus infection. Flow cytometry was used to detect cell surface FV glycosylated gag using the monoclonal antibody mAb34 (Viral antigen). FDCs are B220^+^ cells gated for lack of expression of CD11c, CD8, CD19, CD4, CD3, CD11b and Gr-1, but staining positive for MHCII. The representative plot of FDCs from an acutely infected mouse shows no viral antigen staining above background (Naïve) levels.

One possible explanation for the lack of effect from co-infection with Friend retrovirus was that prion infection and retroviral infection occurred in separate cell populations. Prion replication in the spleen occurs primarily in follicular dendritic cells (FDCs) [26]–[31] and can be detected as early as 1 week post-infection [4]. FV is a gamma retrovirus that requires actively dividing cells for replication but its replication specifically in FDCs has not been investigated. In order to determine if FV infected spleen FDCs during the acute stage of infection, mice were inoculated i.v. with FV and the spleens harvested around the time of peak FV replication 7 days later. Spleen cells were analyzed for viral infection by flow cytometric analysis to detect cell surface glycosylated gag using the monoclonal antibody mAb34 combined with a set of markers to identify FDCs. FDCs have a unique cell surface profile and express the B cell marker B220 but not the B cell marker CD19 or other markers of dendritic cells, monocytes, or T cells [32]. Thus, cells were analyzed by multiparameter flow cytometry to isolate FDCs and determine whether they were infected. During the acute phase of FV infection, viral antigen was not detected above background levels in FDCs but was primarily associated with B220 negative erythroid cells (Figure 6, oval). These data suggest that there was no direct effect of FV on PrP^Sc^ levels because retroviral and prion replication were not occurring in the same cell type.

## Discussion

Co-infection of mouse scrapie infected cells with F-MuLV increased the release of both PrP and scrapie infectivity in vitro (Fig. 1), suggesting that in vivo co-infection might increase cell-to-cell spread of prions and thus influence disease progression. In the early stages of prion infection, both the prion agent and virus would replicate in the spleen with the prion agent eventually spreading from the periphery into the central nervous system via peripheral nerves. We hypothesized that, by increasing the amount of PrP and infectivity released by infected cells, the presence of virus in the spleen would enable a more rapid spread of 22L into the brain and a shortening of prion disease incubation times. However, our data show that co-infection of mice with both FV and 22L has no effect on the outcome of scrapie infection.

Our results are consistent with an earlier study where Friend virus was used to infect mice infected with scrapie [19]. However, because FV causes leukemia in susceptible mice, these earlier studies used mice that had been infected with FV a full two months after being infected with scrapie. Thus, it was still possible that FV had a minimal effect on disease progression because it was not present early during mouse prion infection when efficient replication in the spleen can be important for prion neuroinvasion and spread to the brain [6], [7], [9]. However, our data clearly show that, even when both scrapie and retroviral replication are occurring concurrently in the spleen throughout the course of infection, FV has no discernible effect on scrapie pathogenesis.

The effect of viral co-infection on prion infection can be direct or indirect. Co-infection of prion-infected NIH 3T3 cells in vitro with either Moloney murine leukemia virus [14] or F-MuLV (Figure 1) can lead to an increase in PrP levels as can co-infection of sheep scrapie-infected microglial cells with a sheep lentivirus [15]. Furthermore, the titer of endogenous murine retroviruses in neuroblastoma or hypothalamic cells lines can be increased or decreased when the cells are also infected with prions [33]. Other studies have shown that the level of endogenous retroviruses in the CNS [34], [35] or spleen [36] can be altered by scrapie infection of mice or BSE infection of non-human primates [37]. These data all suggest that, when present in the same cell type, prions can influence retroviral replication and retroviruses can influence prion replication. However, we found no evidence of FV replication in FDCs which are the spleen cell population infected by prions in vivo. It remains possible that co-infection of FDCs by other viruses could modify prion infection.

Retroviral co-infection could also have had indirect effects on prion disease. Follicular dendritic cells are reticular network-forming cells in lymphoid follicles that retain antigen for long periods of time for presentation to B cells, but little is known about their ontogeny and whether they simply migrate to follicles or divide there. Lymphotoxin α/β secreted by B lymphocytes activates FDCs [38] and activated FDCs are important sites of prion replication [26], [30], [31]. It has been suggested that lymphotoxin α/β-triggered differentiation of FDCs is a mechanism by which inflammation could cause prions to replicate in tissues not normally associated with prion infection [12]. Thus, it was also possible that FV infection could indirectly influence prion disease progression via lymphotoxin α/β activation of FDCs. This could lead to increased levels of PrP^Sc^ in the spleen during both the acute and chronic phases of FV infection potentially decreasing prion disease incubation times. However, we observed neither a change in prion disease incubation time nor an increase in the levels of FDCs or PrP^Sc^ in the spleens of infected mice. Our results suggest that, at most, any indirect effect of FV on prion disease in vivo is either too minor or too transient to have an impact on progression to clinical disease.

Our data demonstrating that inflammation in the spleen does not alter prion disease incubation times, brain pathology, or PrP^Sc^ levels are consistent with other studies showing that inflammation in peripheral organs, while it can affect the tissue distribution of prion infectivity, does not affect the normal progression of prion disease or lead to early death in mice [11], [13]. By contrast, intracerebral inoculation of mouse adenovirus at various times post-scrapie infection significantly reduced disease incubation times, albeit without apparently altering scrapie pathology [39]. Furthermore, inducing inflammation in the brain early during prion infection led to a rapid and fatal neurological disease even though PrP^Sc^ levels were not affected [40]. In this latter instance, the acceleration of neurological disease in scrapie-infected mice was likely due to the host response to acute inflammation in the CNS rather than to an increase in prion replication [40]. When taken together with the current study, the data suggest that prion infected mice are much more susceptible to the detrimental effects of viral co-infection of or damage to prion-infected CNS tissues rather than to prion-infected peripheral tissues.
