# Correction: Co-Infection with the Friend Retrovirus and Mouse Scrapie Does Not Alter Prion Disease Pathogenesis in Susceptible Mice

**DOI:** 10.1371/annotation/c6b9e78a-451b-4a34-bcd3-24a820bfa32f

**Published:** 2012-06-12

**Authors:** Pascal Leblanc, Kim Hasenkrug, Anne Ward, Lara Myers, Ronald J. Messer, Sandrine Alais, Andrew Timmes, Suzette A. Priola

There was an error in the last author's name. Suzette A. Priola is correct. 

